# The Effect of Chitosan on Physicochemical Properties of Whey Protein Isolate Scaffolds for Tissue Engineering Applications

**DOI:** 10.3390/polym15193867

**Published:** 2023-09-23

**Authors:** Martyna Gaweł, Patrycja Domalik-Pyzik, Timothy E. L. Douglas, Katarzyna Reczyńska-Kolman, Elżbieta Pamuła, Kinga Pielichowska

**Affiliations:** 1 Department of Biomaterials and Composites, Faculty of Materials Science and Ceramics, AGH University of Krakow, 30-059 Kraków, Poland; m.hunger@wp.pl (M.G.); pdomalik@agh.edu.pl (P.D.-P.); kmr@agh.edu.pl (K.R.-K.); pamula@agh.edu.pl (E.P.); 2 School of Engineering, Lancaster University, Lancaster LA1 4YW, UK; t.douglas@lancaster.ac.uk

**Keywords:** whey protein isolate, chitosan, tissue engineering, hydrogel, scaffold

## Abstract

New scaffolds, based on whey protein isolate (WPI) and chitosan (CS), have been proposed and investigated as possible materials for use in osteochondral tissue repair. Two types of WPI-based hydrogels modified by CS were prepared: CS powder was incorporated into WPI in either dissolved or suspended powder form. The optimal chemical composition of the resulting WPI/CS hydrogels was chosen based on the morphology, structural properties, chemical stability, swelling ratio, wettability, mechanical properties, bioactivity, and cytotoxicity evaluation. The hydrogels with CS incorporated in powder form exhibited superior mechanical properties and higher porosity, whereas those with CS incorporated after dissolution showed enhanced wettability, which decreased with increasing CS content. The introduction of CS powder into the WPI matrix promoted apatite formation, as confirmed by energy dispersive spectroscopy (EDS) and Fourier transform infrared spectroscopy (FTIR) analyses. In vitro cytotoxicity results confirmed the cytocompatibility of CS powder modified WPI hydrogels, suggesting their suitability as cell scaffolds. These findings demonstrate the promising potential of WPI/CS scaffolds for osteochondral tissue repair.

## 1. Introduction

Tissue engineering (TE) has been recently developed as the most effective group of methods for repairing injured tissues. This may overcome the barriers arising from conventional approaches related to tissue regeneration [1]. Allografts face multiple issues, such as compatibility of recipient and donor, limited number of donors, risk of transplant rejection, immunological reaction, pain, or a need to take immunosuppressive drugs [2]. The use of biocompatible scaffolds allows cells to adhere and proliferate, thus facilitating self-regeneration of tissue [3]. Natural materials, especially polymers, have been widely investigated as substrates for tissue engineering. The human tissue-like properties of many natural polymers are desirable for developing the most compatible solutions for tissue loss treatment [4].

Osteochondral tissue (OCT) is composed of articular cartilage, osteochondral interface, and an underlying subchondral bone, which transforms into the trabecular bone. Going further, cartilage consists of another three different layers, dependent on the orientation of the collagen fibers. OCT is a complex heterogeneous tissue with differences in the bioactivity of each zone [5]. Moreover, cartilage is an avascular and aneural tissue. This significantly limits its self-healing abilities. The gradient of all properties between bone and cartilage constitutes a significant problem for the simultaneous regeneration of these two different tissues. One of the most important parameters of OCT scaffolds is microstructure. This significantly influences cell adhesion and proliferation, as well as favors angiogenesis [6,7,8]. Mechanical properties of the scaffolds are, similarly to porosity and biological properties, highly important and should not be overlooked. Appropriate mechanical stimuli have to be transmitted to the newly formed tissues. Otherwise, the reconstructed tissue will not have the appropriate functionality and may generate new defects [9]. Biodegradability is another crucial property which should be specified and adjusted to the regeneration and reconstruction time of OCT. Important properties that are considered in TE studies are water absorption and wettability. The ability of the material to absorb water and its surface properties are essential for cell adhesion, thus influencing OCT regeneration [10].

OCT engineering approaches have been significantly focused on hydrogel scaffolds, for example protein-based [11] or polysaccharide-based [12,13] biomimetic scaffolds. However, the suitable natural hydrogel-based complex scaffold with desirable complete long-lasting functionality has not been developed so far. Many different forms of natural hydrogel modification to improve its properties have been known. The mixing, formation of composites or the addition of bioactive molecules are often reported in the field of OCT tissue engineering [14].

Natural polymers are mainly derived from sources such as plants, animals, or microorganisms [4]. ‘Green’ technology has become more popular recently, therefore combining food waste industry and tissue engineering approaches may be an ecological way to develop effective methods of tissue regeneration. Moreover, using food waste as materials for biomedical applications may contribute to decreasing the negative influence on the environment and the economy. Processing food, its residues, and byproducts may generate a valuable source of bio-substitutes. One of the widely used polymers in TE is chitosan—a derivative of chitin, extracted from crab and shrimps shells. Another example is that of whey proteins, as a by-product of the dairy industry [15,16,17].

Whey protein isolate (WPI) is a specific, water-soluble compound of whey, which is produced during cheese manufacturing. It is formed as a result of the precipitation of proteins in milk facilitated by microorganisms. As the highest form of purification, WPI consists of >90% protein, lactose, fat, ash and approximately 4–5% of moisture [18]. β-lactoglobulin (β-LG) and α-lactalbumin (α-LA) are usually the main components, next to glycomacro-peptide (GMP), immunoglobulins (Igs), bovine serum albumin (BSA) and others. Although it is considered as an environmental pollutant, WPI has many advantages that make the material a potential application not only in food products but also in tissue engineering as a biomaterial [19]. The amount of β-LG in WPI is in the range of 45–90% [20,21,22,23,24,25,26]. Its structure containing two disulfide bridges and one free thiol allows the formation of a hydrogel network under high pressure or temperature by thermal denaturation. This suggests that different types of WPI are obtained, depending on β-LG amount.

The application of WPI-based hydrogel scaffolds in tissue engineering is a niche in scientific research. There is a limited number of studies related to this field. Dziadek et al. [27] developed novel multicomponent WPI-based hydrogel composites for bone regeneration. Gelatin powder of 20 wt.% was added to 40% WPI aqueous solution, then mixed with α-TCP (0–70 wt.%) dispersion. Crosslinking was carried out at 100 °C and autoclaved for 30 min at 121 °C, thus providing simultaneous sterilization. The tested systems showed a significant improvement in mechanical properties, high bioactivity in vitro in simulated body fluid (SBF), and cytocompatibility, thus enhancing osteoblast-like cells to adhere, spread and proliferate. Gupta et al. [28] proposed 40% WPI aqueous solution with CaCO_3_ to improve porosity and bioactivity of the hydrogel. Autoclaving at 120 °C for 2 h allowed effective crosslink and sterilization of the materials. The composites provided concomitantly higher porosity and mechanical properties. Pores larger than 100 μm in diameter were obtained and osteoblast-cell proliferation was also supported. The products of degradation were not cytotoxic. There is also some evidence that WPI acts as an antioxidant and antibacterial agent. Carson et al. [29] investigated antimicrobial activity of complexes based on polyphenols from green tea extract and WPI. The obtained results confirmed antioxidant and antibacterial activity of the complexes against Gram-positive bacteria. Rouabhia et al. [30] conducted in vivo evaluation in mice of WPI-based and pure β-LG-based films as dermal scaffolds, which were found to be non-toxic and nonimmunogenic. Furthermore, the films were biodegradable—signs of early degradation, such as crumbling or erosion, were observed after 15 days of implantation.

Chitosan (CS), as the second most abundant polysaccharide, after cellulose, has been widely investigated. It has been used in many areas, especially the biomedical, due to its preferable properties, such as excellent biocompatibility, nontoxicity, biodegradability, and antimicrobial activity. CS is very susceptible to various chemical and physical modifications, which makes it very often used as a hydrogel in tissue engineering [31,32,33].

Many reports related to chitosan-based hydrogels as scaffolds for tissue engineering have been published over the last few decades [34,35,36,37,38]. Its similarity to glycosaminoglycans present in the cartilage extracellular matrix plays an important role in osteochondral tissue engineering [39]. Shamekhi et al. [40] presented hydro-thermally crosslinked chitosan (medium molecular weight and deacetylation degree (DD) 75–85%), with simultaneous sterilization using an autoclave. The process is based on the reaction between the amine and carbonyl groups, known as the Maillard reaction. The wide range of methods confirmed an improvement of CS physical and mechanical properties, as well as showing no cytotoxicity of the CS porous scaffolds. Autoclaving provided CS with higher chemical stability—mass loss was lower at about 10–20% after 12 weeks in lysozyme solution. Moreover, the viability of human chondrocytes was confirmed after 7 days of cell culture by MTT test. Jin et al. [41] developed injectable chitosan-based hydrogels crosslinked by enzymatic reaction as a support for cartilage regeneration. The in vitro showed the viability of round-shaped cells after 2 weeks. CS is very often modified to improve or add specific properties. Yu et al. [42] combined genipin-crosslinked chitosan hydrogel with hydroxyapatite (HAp) and graphene oxide, thus obtaining high strength scaffold for bone tissue engineering. The material showed an oriented structure with high porosity and good biocompatibility. HAp may also be considered as a bioactive agent [43]. Demirtaş et al. [44] developed an injectable thermally crosslinked chitosan-based hydrogel. Pure CS (DD = 75–85%) and that loaded with HAp and osteoblasts suspensions were prepared. Mineralization and osteogenic differentiation were observed after 21 days of cell culture. Additionally, HAp significantly improved the mechanical properties, which is desirable for chitosan as a scaffold for bone regeneration. There are also known functionalized forms of CS for obtaining a mechanically strong photopolymerizable form of CS-based hydrogels. An addition of collagen to the CS matrix was described in the work of Arakawa et al. [45]. This provided slower biodegradation and significantly enhanced osteoblasts’ adhesion and proliferation. Scaffolds based on chitosan and collagen are well-known in bone and cartilage tissue engineering. A combination of these two polymers allows the tailoring of properties toward both bone and cartilage [46]. There are also bi-layered CS-based systems, which may be an effective way to repair osteochondral loss. Malafaya et al. [47] used nonmodified CS (DD = 85%) and HAp-modified CS to obtain particles crosslinked with glutaraldehyde. After crosslinking, the layers were laid and pressed into cylindrical molds, then dried at 60 °C for 3 days. The studies suggest a cytotoxic behaviour of composite materials due to unsintered HAp, while using sintered HAp leads to no cytotoxic behaviour. However, there is some evidence that glutaraldehyde as a crosslinking agent may cause such a reaction [48].

A new solution to effectively repair osteochondral tissue may be a combination of WPI and CS. There are reports describing the Maillard reaction between protein and polysaccharide [49]. Wang et al. [50] studied covalent interactions between WPI and inulin as a polysaccharide (mass ratio 1:1; aqueous solution). FTIR measurements confirmed a decrease in the number of free amino acid groups and the formation of new C-N bonds. Moreover, the increased antioxidant activity of inulin-modified WPI conjugates was observed. Miralles et al. [51] also presented a beneficial influence of CS addition on the biological properties of β-LG. CS and WPI have opposite charges at pH > 5.3, therefore electrostatic interactions are possible, and thus formation of a complex. Moreover, heating may improve such interactions [52,53]. Zheng et al. [54] confirmed a beneficial influence of CS addition on WPI properties, such as heat stability or emulsifying properties. Yang et al. [55] formed thermo-gels by heating CS/WPI/wheat starch dispersions at 85 °C for 75 min. Previously, the pH of the solutions was adjusted to 5.5 to provide the most effective cross-linking. The medium molecular weight CS provided better chemical stability protection for the tested systems, compared to the low molecular weight chitosan. Hu et al. [56] also confirmed the improved stability and antioxidant activity of the CS/WPI fibrils complex with an increase in the amount of CS in comparison to pure WPI. The effective interactions between CS and WPI make these materials very interesting to apply together as a hydrogel scaffold for osteochondral tissue engineering.

The main goal of this study was to obtain natural hydrogels based on WPI and CS, as well as to evaluate the impact of the polysaccharide addition on the physicochemical and biological properties of the WPI-based hydrogel. The optimal chemical composition of the hydrogel was chosen based on physicochemical and biological properties: morphology, structural properties, chemical stability, swelling ratio, wettability, mechanical properties, in vitro bioactivity, and cytotoxicity assay.

## 2. Materials and Methods

### 2.1. Materials and Samples Preparation

Medium molecular weight (M = 100,000–300,000 g/mol) chitosan (CS) was purchased from Acros-Organics (Morris Plains, NJ, USA); whey protein isolate (WPI) with 97.7% protein and 75% β-LG (according to the specification provided by the manufacturer) was obtained from Davisco Foods International, Inc. (Eden Prairie, MN, USA). The reagents necessary for the preparation of the phosphate buffered saline (PBS) and simulated body fluid (SBF) were obtained from Avantor Performance Materials Poland S.A., Gliwice, Poland. All the reagents were used as received.

Two types of the WPI-based materials modified by CS addition were prepared. An appropriate amount of the CS powder was mixed with a fixed amount of WPI powder and then either dissolved in a fixed amount of 2% acetic acid (AcAc, Avantor Performance Materials Poland, Gliwice, Poland) to obtain chitosan solution equal to 1%, 5%, 10%, 20%, and 40% (CSsol), or suspended in deionized water in an amount of 20%, 30%, 40%, 50%, and 60% (CSp). In both groups, the final WPI concentration was set at 40%. The as prepared mixtures were mixed again after 24 h and left for another 24 h to degas. The composition of all the samples is summarized in Table 1; 40% water-based solution of WPI was used as a reference. After this time, they were mixed again (5 min, electric stirrer, manual) to avoid sedimentation of the additives. The resulting mixtures were placed in sealed containers in an oven and crosslinked at 90 °C for 30 min to ensure complete denaturation of the protein.

### 2.2. Characterization

The microstructure and morphology of the samples were studied using an optical microscope (VHX-900F, Keyence, Mechelen, Belgium) and a scanning electron microscope (SEM, Nova NanoSEM 200, FEI, Eindhoven, The Netherlands) equipped with an energy dispersive X-ray (EDX) analyzer (EDAX Company, Pleasanton, CA, USA) at 5 kV electron beam energy with magnification 500–10,000×. EDX analysis was performed at several randomly selected points and as average analysis of the observed surface. All samples were coated with a carbon layer prior to the measurement. Porosity was assessed using ImageJ v. 1.8.0 software. The scaffolds were freeze-dried and cut into thin slices prior to the analysis.

The chemical structure of the materials was identified using Fourier-transform infrared spectroscopy in the attenuated total reflection mode (FTIR-ATR). The spectra were taken after 64 scans, with the resolution of 4 cm^−1^, in the range of 4000–600 cm^−1^ on Bruker Tensor 27 (Bruker, Poznań, Poland) with a diamond crystal. The results were obtained and modified using OPUS Spectroscopy Software 8.5 and SpectraGryph 1.2, respectively.

A universal testing machine (Zwick 1435, ZwickRoell GmbH & Co. KG, Ulm, Germany) with a 5 kN load cell was used to assess mechanical properties in the compression test (3 mm deformation; 2 mm/min test speed). All measurements were made for 6 mm cubic scaffolds at room temperature. Young’s modulus was determined based on a linear fragment of the stress–strain curve. At least three independent measurements were averaged for each type of sample.

To characterize swelling properties, the cubic samples (3 per material type) were weighed and then immersed in PBS (pH = 7,4, T = 37 °C). The swelling ratio was evaluated after 1, 2, 3, 4 and 5 h of the incubation, and then day by day during the 10 following days. At these time points, the swollen samples were taken out, carefully dried with a tissue paper, weighed, and then placed back in the PBS solutions. The swelling ratio (*SR*) was calculated according to Equation (1):

(1) $$SR=\frac{M_{i}-M_{0}}{M_{0}}\;\cdot \;100\;\left[\%\right]$$

where: *M_i_*—the weight of the swollen sample after drying with tissue paper [g], *M*_0_—the initial mass of the sample [g].

The measurement of the water contact angle (WCA) was used to assess the scaffold’s wettability. Using a goniometer (Drop Shape Analyzer, DSA 10, KRÜSS GmbH, Hamburg, Germany), at room temperature, a sessile drop of deionized water was put onto the surface of the materials and the WCA of the materials was determined based on at least six independent measurements.

The degradation process was carried out in PBS solution at 37 °C (pH = 7.4). The proportion of sample mass to the volume of PBS was 1 g:100 mL. The samples were weighed, placed in the solution, and incubated at 37 °C for 1, 2, 3, 4, 5, 6, 7 days and then weekly for up to 12 weeks. At these intervals, pH of the solution was measured. PBS was refreshed weekly. After 3 months of incubation, all samples were weighed. The weight loss (*WL*) was calculated using Equation (2):

(2) $$WL=\frac{M_{0}-M_{j}}{M_{0}}\;\cdot \;100\;\left[\%\right]$$

where: *M*_0_—the initial mass of the sample [g], *M_j_*—the weight of the samples after 12-week incubation [g].

The preliminary bioactivity assay was performed using the improved SBF protocol described by Bohner et al. [57]. The samples were weighed, immersed in the SBF (with mass to volume ratio 1:50) and kept at 37 °C for 2 and 4 weeks. After each time point, the samples were taken out, gently washed with deionized water, and freeze-dried. SEM-EDS and ATR-FTIR were used to determine any changes caused by the SBF incubation.

Based on the results of the physicochemical characterization, for the cell culture study the following samples were chosen as reference: CSsol20, CSsol40, CSp20, CSp40 and WPI_0. Cytotoxicity evaluation was carried out using extracts in accordance with the ISO 10993-5 standard [58]. The cell culture was carried out with the MG-63 cell line (European Collection of Cell Cultures, Sailsbury, UK). Cells were cultured in EMEM (Eagle’s Minimal Essential Medium, PAN BIOTECH, Aidenbach, Germany) with the addition of 10% fetal bovine serum (FBS, Biowest, Nuaillé, France) and 1% antibiotics (penicillin/streptomycin, PAA, Pasching, Austria), 0.1% amino acids and 0.1% pyruvate (PAA, Pasching, Austria). The culture was conducted at 37 °C, 5% CO_2_ and under increased humidity.

Extraction was prepared based on the mass of the tested material to the volume of the culture medium ratio equal to 100 mg:1 mL. The freeze-dried samples were weighed and immersed in EMEM, then left for 24 h at 37 °C. The extracts were sterilized by filtration using syringe filters (0.22 µm). The following dilutions of the extracts were prepared using EMEM: 1 (undiluted), 1/2, 1/4, 1/8 and 1/16. EMEM maintained under conditions identical to the tested samples was used as a control.

Cells were seeded in 96-well plates at 5000 cells per well (100 µL medium). After 24 h of incubation, the medium was replaced with the appropriately diluted extracts (100 μL) in triplicate. After 24 h, cell metabolic activity and cell viability were tested using the AlamarBlue assay and live/dead staining. For AlamarBlue assay, cell culture medium was withdrawn from all test wells and replaced with 150 µL of fresh EMEM containing 5% AlamarBlue reagent (in vitro toxicology assay kit, resazurin-based, Sigma Aldrich, St. Louis, MI, USA). After 3 h of incubation, 100 µL of medium was transferred into black 96-well plate and fluorescence was measured at λ_ex_ = 544 nm and λ_em_ = 590 nm (BMG Labtech spectrofluorometer, FluoStar Omega, Offenburg, Germany). Percentage resazurin reduction (*RR*) was calculated using Formula (3):

(3) $$RR\;[\%]=\frac{F_{sample}-F_{0\%\;red}}{F_{100\%\;red}-F_{0\%\;red}}\times 100\%$$

where: *F_sample_*—fluorescence of the tested sample, *F*_0%_ *_red_*—fluorescence of culture medium with the addition of AlamarBlue reagent without cells, *F*_100%_ *_red_*—fluorescence of culture medium with the addition of AlamarBlue reagent reduced 100% by autoclaving (15 min, 121 °C). The statistical analysis was performed using ANOVA (One Way Analysis of Variance) followed by Tukey post-hoc test (*p*-value < 0.05). For live/dead staining, a mixture of 0.1% calcein AM and 0.1% propidium iodide (Sigma Aldrich) in PBS was prepared. Cell culture medium was withdrawn from wells and replaced with 100 µL of staining solution. After 20 min incubation in the dark, the cells were observed under Zeiss Axiovert 40 fluorescence microscope with HXP 120C Metal Halide Illuminator (Carl Zeiss, Jena, Germany) at 100× magnification.

## 3. Results and Discussion

**Figure 1:**
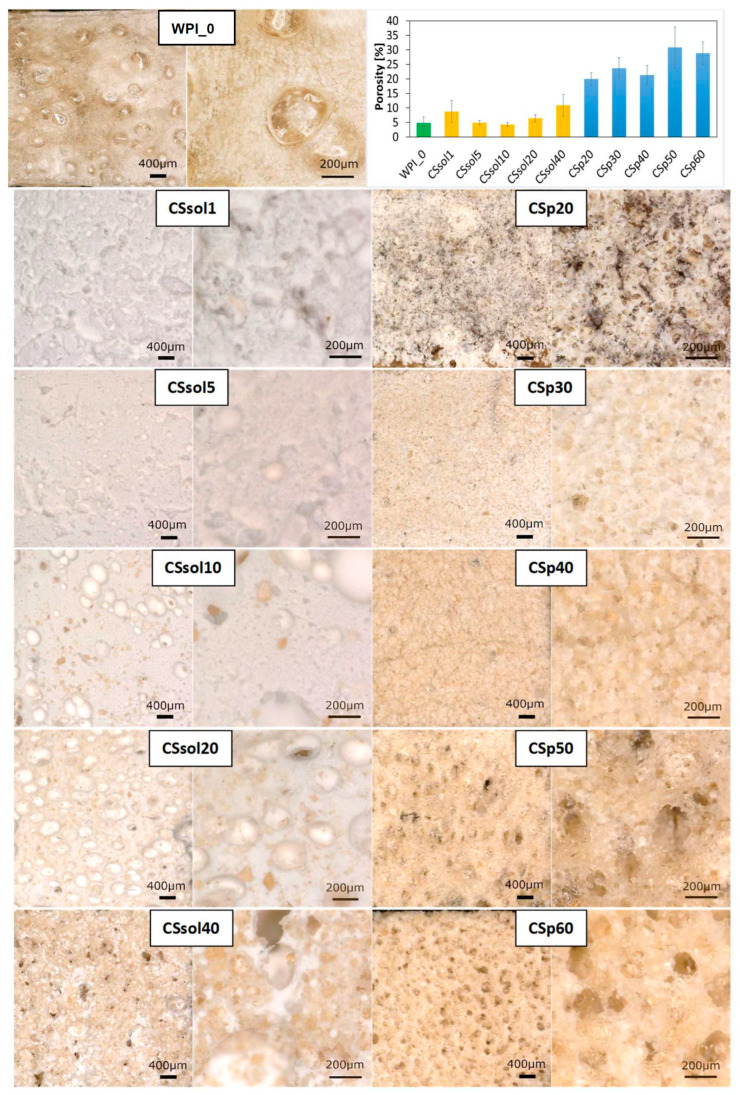
Digital microscopic images (mag.: 50× and 200×): on the top—pure WPI sample; left column—CS solution-based WPI hydrogels; right column—CS powder-based WPI hydrogels; Porosity of the tested samples calculated on a basis of the measurement prepared in ImageJ Software.

Initial observations of the morphology and macro-porosity of the scaffolds were carried out using an optical microscope (Figure 1). It was observed that the microstructure of the samples varied significantly. WPI solution forms a foam after vigorous mixing and the bubbles were visible on the freeze-dried surface. However, no open pores were visible. Both CS solution and CS powder caused the changes in porosity. The more CS in the WPI matrix, the more pores and the larger the pore size observed. The increasing concentration of CS affected the porosity and pore size by forming a denser structure and aggregates [59]. More regular pores were observed for the CS-powder samples. The samples also provided open porosity, which is beneficial for potential nutrient transport to effectively regenerate damaged tissue. CSp30, CSp40, CSp50 and CSp60 had the highest porosity and pore size in the range of 100–200 μm, showing that they might be considered for, e.g., osteochondral tissue engineering [7,60,61].

**Figure 2:**
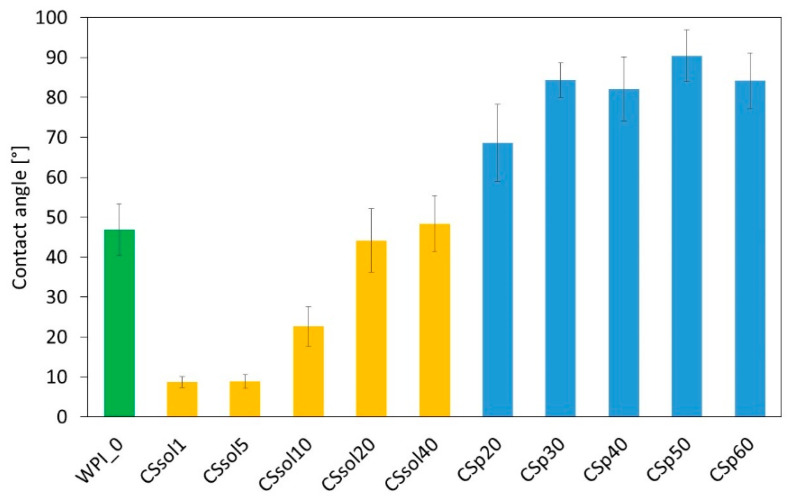
Mean values of water contact angle for all the samples (WPI—whey protein isolate, CS—chitosan).

Another important criterion for TE scaffolds is their wettability. Pure WPI samples were hydrophilic. Depending on the fabrication method, the addition of CS had a different effect (Figure 2). For all the samples with CS powder that was only water-suspended and mixed with WPI, the WCA increased, almost changing the surface character to hydrophobic (WCA > 90). When CS solution was used, especially at lower concentrations (CSsol1, CSsol5, CSsol10), the contact angle decreased, resulting in a very hydrophilic surface.

Researchers in recent years have begun to focus on the surface wettability of hydrogels. It was revealed that the modulation of the surface wettability of hydrogels can overcome some their defects, such as unexpected bacteria adhesion, undesired protein adsorption, easy dehydration in air and swelling in water, and allows enhancement of the hydrogels–cell interactions [62]. The relationship between hydrogel physicochemical parameters and surface wettability is a very complex issue; however, it was observed that the procedure of CS incorporation to WPI strongly affected the wettability of the hydrogels and other physiochemical properties.

**Figure 3:**
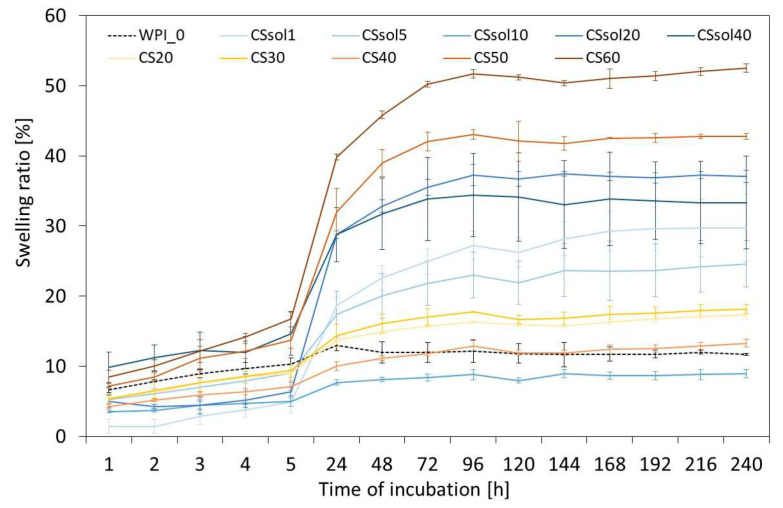
Swelling ratio of the samples calculated up to 240 h of incubation in PBS (37 °C).

In vitro swelling test and chemical stability assay in PBS were carried out to predict the materials’ behavior in physiological conditions. The reference WPI sample had a swelling ratio of about 10% through the entire observation period (Figure 3).

The greatest increase in swelling was noticeable after the first 5 h of incubation, especially for the CSp60 and CSp50 samples (more than 40% after 5 h). In the WPI/CSsol group, the highest swelling was also observed for the samples with the highest addition of CS (CSsol20 and CSsol40). This was probably related to the stronger interactions between the water and chitosan macro-chains.

**Figure 4:**
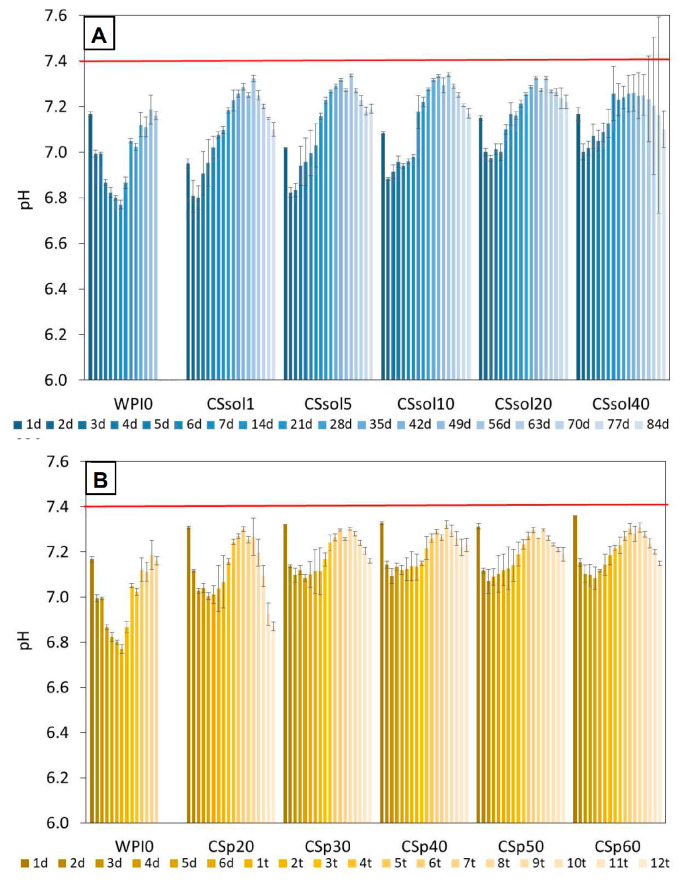
Changes in pH of PBS-based solution during 12 weeks incubation of the samples: (**A**)—CS dissolved, (**B**)—CS powder suspended in water.

The pH measurements of the PBS summarized in Figure 4, showed that the pH during incubation for all samples was in the range of 7.0–7.4, but usually, at the beginning of the incubation, was lower than after a longer time. The lowest pH was found for unmodified WPI. Moreover, a quite similar pH change profile was observed for the sample with dissolved chitosan and for the samples with chitosan added in the form of powder. In general, pH was in the range close to the pH of human blood. Guo et al. [61] observed a similar behaviour of PLLA systems with chitosan [63] where. for the samples with higher chitosan content in the first weeks of incubation, a slight increase in pH was observed, while in subsequent weeks the pH dropped.

**Figure 5:**
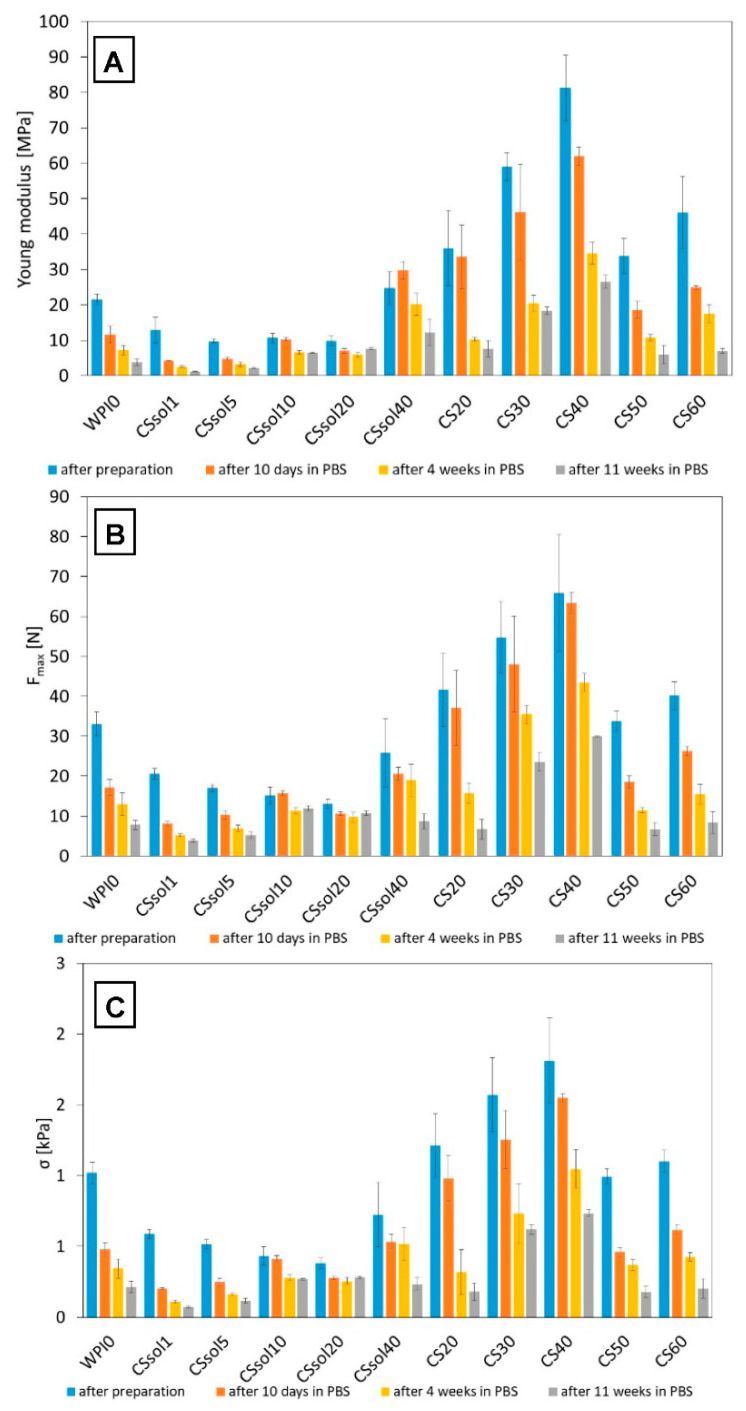
Mechanical properties of the samples after preparation and incubated in PBS: Young’s modulus (**A**), maximum compression force (F_max_) (**B**), and compressive strength (σ) (**C**).

The results of mechanical properties evaluation are summarized in Figure 5. It should be noted that the compositions in which CS solutions were used had lower mechanical parameters than pure WPI. This effect could be attributed to the weak physical interactions between chitosan and WPI macro-chains. The best mechanical properties were found for samples CS30 and CS40, where chitosan was incorporated in powder form. This suggests that chitosan in such concentrations acts as a reinforcement for the WPI matrix. However, further increase in CS content leads to decrease in compressive strength and other mechanical parameters. It is likely that the presence of larger amounts of CS microparticles hinders effective WPI hydrogel thermal crosslinking, because of decreased mobility of WPI macro-chains and their confinement between CS chains. For samples with dissolved CS, it was observed that increasing CS content leads to an increase in compressive strength of the obtained samples. This effect can be connected to the formation of WPI/CS complexes and the formation of stronger intermolecular interactions between WPI and CS macro-chains.

### 3.1. Bioactivity Assay

**Figure 6:**
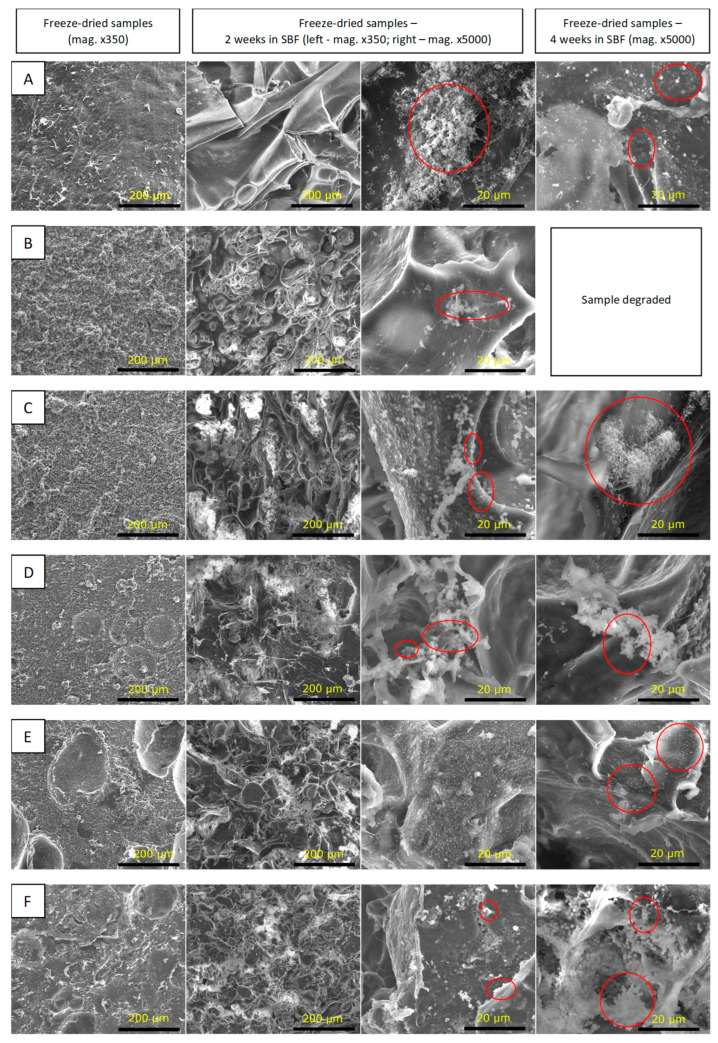
SEM microphotographs of the tested samples before and after incubation in SBF: (**A**)—WPI_0; (**B**)—CSsol1; (**C**)—CSsol5; (**D**)—CSsol10; (**E**)—CSsol20; (**F**)—CSsol40.

**Figure 7:**
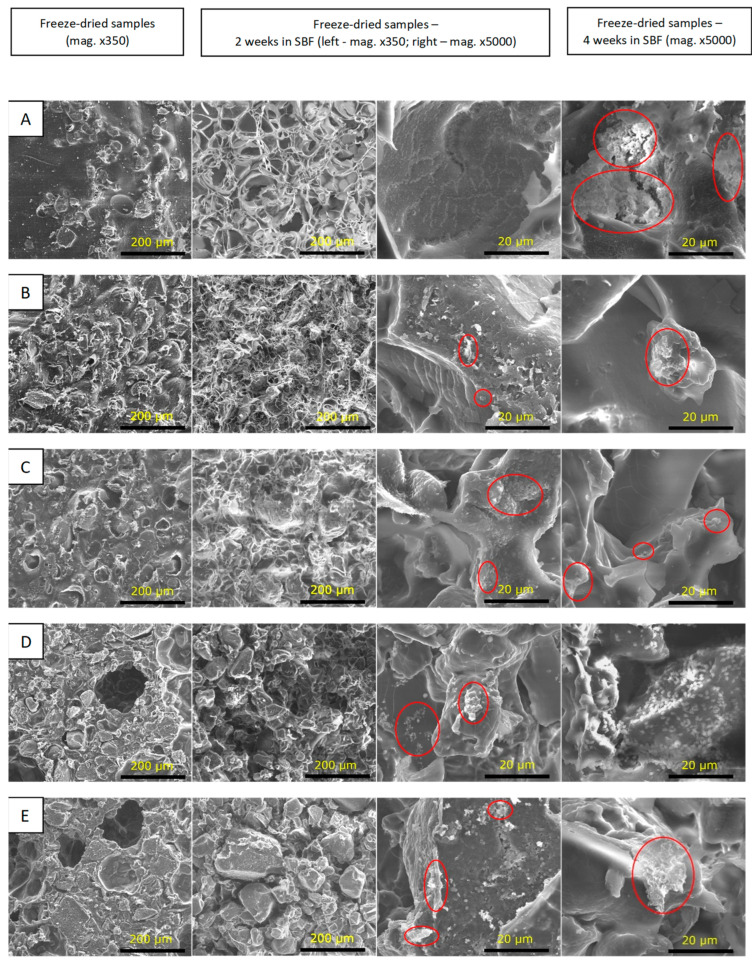
SEM images of the tested samples before and after incubation in SBF solution: (**A**)—CSp20; (**B**)—CSp30; (**C**)—CSp40; (**D**)—CSp50; (**E**)—CSp60.

The SBF incubation test was used to predict possible bioactive properties of the samples based on their ability to form calcium phosphate or apatite-like layers on the surface. Scaffolds intended for use in osteochondral tissue engineering should stimulate bone formation in the area of the subchondral bone. At the same time, they cannot cause calcification in the cartilage zone. Hence, it is necessary to control the mineralization process [64]. SEM images of the CS-solution and CS-powder-based samples are presented in Figure 6 and Figure 7, respectively. All the samples were dimensionally stable after 2 weeks’ incubation in SBF. Only CSsol1 degraded after 4 weeks of incubation. Ineffective interaction between CS and WPI and weakening of the structure may affect the chemical stability of the sample. After 2 weeks of incubation in SBF, the structure was partially degraded and the additional, regular porosity was found. This may enhance cell adhesion and proliferation during the degradation process. It is vital to provide stable properties for the scaffolds, which are beneficial for cell viability during the initial time of tissue defects’ treatment. Particularly, porosity, mechanical properties and bioactivity need to be maintained, so as not to disrupt the process of healing [9].

The incubation in SBF confirmed the preliminary bioactivity of the CS-solution and CS-powder-based samples after 2 and 4 weeks of incubation. Cauliflower-like structures of apatite (calcium phosphates, CaP) formation was observed for CSsol5, CSsol10, CSsol40 and CS-powder-based samples.

**Figure 8:**
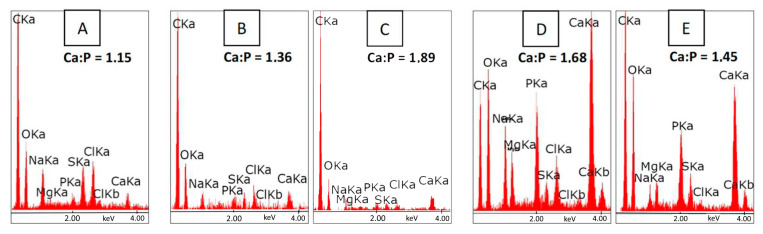
EDS analysis for the representative samples after 4 weeks in SBF: (**A**)—WPI_0; (**B**)—CSsol20; (**C**)—CSsol40; (**D**)—CSp20; (**E**)—CSp40.

The introduction of CS powder to the WPI matrix promoted apatite formation, which was confirmed by the EDS analysis (Figure 8). Moreover, the number of apatite crystals increased after 4 weeks. The surface of the pure WPI sample was covered by a thick layer of CaP after 2 weeks in SBF. However, after 4 weeks, a thinner layer was observed. The lower chemical stability may cause the detachment of CaP molecules. As chitosan supports scaffold mineralization [65], its increased amount in the WPI structure may provide higher osteo-conductivity of the material. The EDS analysis also shows the presence of chloride and magnesium, which are the residues of the SBF solution. Sulfur peaks were also observed as a proof of the presence of WPI. This suggests that the sulfur bridges present on the WPI surface are stable despite prolonged immersion in the SBF. EDS measurement allowed evaluation of Ca:P ratios to assess the similarity of the structures formed on the surfaces to the human bone, where the stoichiometric hydroxyapatite occurs (Ca:P = 1.67) [66]. On the WPI surface, calcium-deficient HAp formation was observed (Ca:P = 1.15), while non-stoichiometric HAp was found on CSp40, CSsol20 and CSsol40. However, CSsol40 supported formation of the phosphorus-deficient HAp (Ca:P = 1.89). The highest similarity to the HAp in the human bone had an apatite-covered CSp20 surface (Ca:P = 1.68), which may provide the most efficient regeneration of bone region in OCT. Moreover, this sample supports formation of the larger amount of apatite.

**Figure 9:**
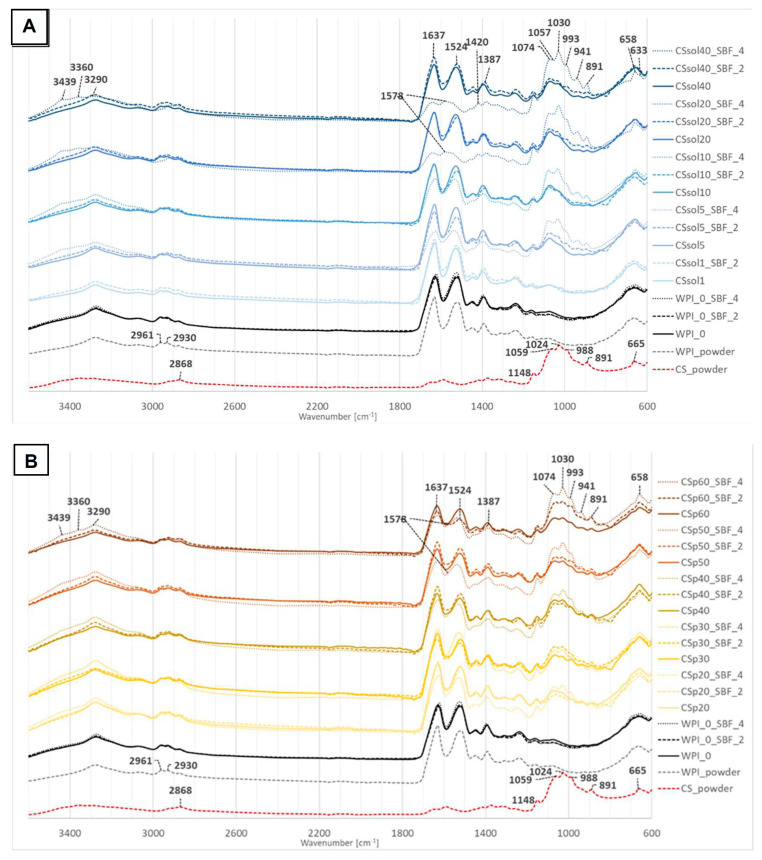
FTIR spectra of freeze-dried, modified and non-modified WPI-based samples—before, after 2 and 4 weeks in SBF: (**A**)—CS solution-based samples, (**B**)—CS powder-based samples.

The characteristic absorption bands for CS and WPI powders, as well as for the tested hydrogels, are presented in Figure 9A,B, for CS solution and CS powder-based samples, respectively. FTIR spectra for the samples after 2 and 4 weeks of incubation in SBF are also shown.

There was no significant difference between WPI powder and WPI_0 hydrogel. The characteristic absorption band at 3270–3290 cm^−1^ related to the stretching vibrations of hydroxyl, and –NH_2_ groups were observed. The triple band in the region of about 3050, 2930 and 2961 cm^−1^ corresponds to –CH_2_ stretching of aliphatic groups, both aromatic and aliphatic. The primary structure of WPI was confirmed by the intense absorption band at 1637 cm^−1^ and is related to amide groups –CO-NH_2_. Absorption bands at 1524 and 1230 cm^−1^ were attributed to the secondary and tertiary amide groups of WPI. The presence of carbonyl and –C-O-H groups was confirmed by the band at 1387 cm^−1^ and the small band at about 1060 cm^−1^ [67,68]. In CS spectrum, a broad band between 3000–3500 cm^−1^ corresponding to stretching vibrations of–OH and –NH groups was found, while a small signal at 2868 cm^−1^ confirmed the presence of –CH groups. The double band in the range of 1570–1670 cm^−1^ is attributed to carbonyl groups C=O and –NH bonds, which represent the primary and the secondary amide structure, respectively. CH_3_ wagging vibrations resulted in the band at about 1410 cm^−1^. A small peak at about 1380 cm^−1^ confirmed the glucosamine groups consisting of the C-C stretching vibrations. Amine I, amine II and amine III were visible in this region. The multiple intense peaks in the range of 891–1059 cm^−1^ correspond to C-O stretching vibrations and represent the saccharide structure of CS [69,70,71]. No shifts and structural changes were observed after thermal crosslinking in the WPI structure and the addition of CS.

For the SBF-incubated samples, specific regions related to apatite formation were observed. A partial degradation of the WPI structure and the formation of the apatite layer can be confirmed by the reduced doublet band in the range 1524–1637 cm^−1^ and the presence of the intense multiple bands at 941, 1030, 1074 cm^−1^, respectively. The triple band corresponds to the asymmetric vibrations of –PO_4_ groups, while the small peak at 1420 cm^−1^ is related to the stretching vibration of –CO_3_ groups of carbonated apatites, thus suggesting apatite formation on the hydrogels’ surface. The symmetric vibrations of the -PO_4_ groups result in the presence of the band at 941 cm^−1^. Moreover, the specific signal of hydroxyl groups at 3439 cm^−1^ was observed. A small band at 633 cm^−1^ appeared and corresponds to the –OH groups as a result of water absorption [72,73]. The changes related to apatite formation are the most pronounced in the samples with the highest amount of CS added.

However, the bioactivity-related results should be verified in the in vivo conditions, where the actual biological phenomena can be tested and thoroughly explained.

### 3.2. Cytotoxicity Evaluation

**Figure 10:**
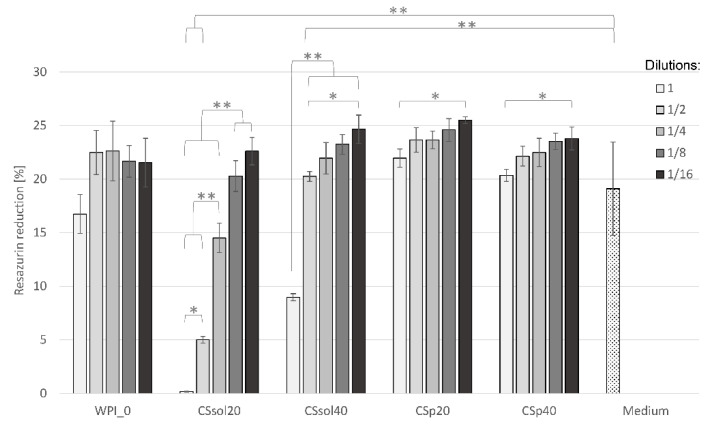
Metabolic activity of the MG-63 cells cultured for 24 h with the samples extracts (1, 1/2, 1/4, 1/8, 1/16 denote the dilutions) and undiluted medium. Statistically significant differences at * *p* < 0.05 and ** *p* < 0.01 (one-way ANOVA followed by Tukey post-hoc test).

Based on the results of the physicochemical characterization, only CSsol20, CSsol40, CSp20, and CSp40 were selected for preliminary cytotoxicity screening. MG-63 cells were cultured in the materials extracts and their dilutions. The results were compared to those of WPI and the medium used as control. The samples containing only WPI (WPI_0) did not influence cell viability regardless of extract dilution (Figure 10), which confirm that WPI is cyto-compatible with MG-63 cells. Similar materials based on WPI have already been tested by Plastun et al. [74] on L929 fibroblasts and by Norris et al. [75] on MC3TC-E1 cells. Both studies found out that WPI-based materials were not cytotoxic against different cell types. With regard to the culture in a pure medium, metabolic activity of the cells measured as percentage resazurin reduction was at a similar level within the margin of error for all tested hydrogels except for undiluted extracts of CSsol20 and CSsol40 and twice diluted extracts of CSsol20. In the case of CSsol20 samples, almost no metabolic activity was detected for undiluted extracts, ½ dilution allowed the survival of around 25% of cells in comparison to control, while further dilutions resulted in decreased cytotoxicity (i.e., 1/4 dilution was not found to be statistically significant from control, but cell viability was reduced, in comparison to 1/8 and 1/16 dilution). Undiluted extracts from CSsol40 samples also exhibited significant cytotoxicity against MG-63 cells, nonetheless ½ dilution was sufficient to completely reduce toxicity.

On the contrary, even undiluted extracts from CSp20 and CSp40 samples were not found to be cytotoxic in comparison to untreated cells or WPI0. This suggests that addition of chitosan in a form of powder (without dissolution in acetic acid) was favorable in terms of cytocompatibility of the obtained materials. Observed cytotoxicity in CSsol samples may be attributed to residual acetic acid being released to the cell culture medium causing the decrease in medium pH. Although no significant differences between CSsol and CSp samples were found during incubation in PBS (Figure 4), we believe that different compositions of cell culture medium containing 10% fetal bovine serum might lead to more efficient dissolution of acetic acid remnants. Use in a powder form not only improved mechanical properties and bioactivity of the scaffolds, but also made them cyto-compatible with MG-63 cells.

**Figure 11:**
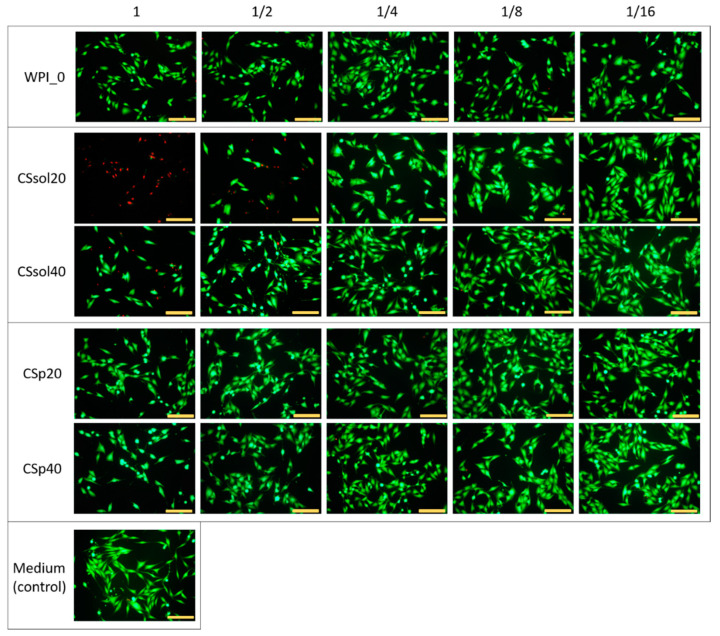
Live/dead fluorescent staining of the MG-63 cells cultured for 24 h with the sample extracts (1, 1/2, 1/4, 1/8, 1/16 denote the dilutions). Scale bar: 200 µm.

Fluorescent live/dead staining (Figure 11) confirmed the presence of viable cells in the case of almost all samples and dilutions. Dead cells were particularly visible for undiluted (almost 100%) and diluted by half CSsol20 (around 45%) extracts, and some were also present in the undiluted CSsol40 sample (around 43%). In both WPI/CS-powder-based extracts, a large number of viable cells was visible, with less than 2% dead cells observed in the field of view. The morphology of viable cells in all samples was similar to control, and the cells were well spread on the surface. The majority of the cells exhibited an elongated shape characteristic of MG-63 cells.

Preliminary cytotoxicity studies showed that CS-modified WPI hydrogels (especially those containing CS in a powder form) were not cytotoxic for MG-63 cells, and considering their physico-chemical properties, they may be considered as suitable candidates for osteochondral scaffolds. To fully assess their potential in the field of tissue engineering, further studies, including evaluation of cytocompatibility with human mesenchymal stem cells and chondrocytes in direct contact with materials (cell viability, proliferation) and the influence of scaffold composition and morphology on cell differentiation, are needed.

## 4. Conclusions

Whey protein isolate and medium molecular weight chitosan were combined to create hydrogels with possible application in osteochondral tissue engineering. The effect of the amount and the form of chitosan (solution vs. powder) was evaluated. Chitosan was shown to positively affects WPI properties, such as chemical stability, bioactivity, and cytotoxicity. The highest porosity with more regular pores was found for the samples created with the use of CS powder, whereas the wettability investigation showed that, when the CS solution was used, especially at lower concentrations, contact angle decreased, resulting in a very hydrophilic surface. The largest increase in swelling was revealed for the CSp60 and CSp50 samples and CSsol20 and CSsol40. The increased swelling for the samples with the highest CS content was probably related to the stronger interactions between the water and chitosan macro-chains. The best mechanical properties were observed for the samples CS30 and CS40, where chitosan was incorporated in the powder form, suggesting that chitosan microparticles can act as reinforcement for the WPI matrix. Introduction of the CS powder into the WPI matrix promoted apatite formation, that indicates the potential bioactivity of the samples modified with CS. In vitro cytotoxicity studies confirmed that CSp-modified WPI hydrogels are not cytotoxic and are promising candidates for osteochondral scaffolds. All obtained results show the great potential of WPI/CS scaffolds in biomedical applications.

## Figures and Tables

**Table 1:** Description of the WPI/CS systems tested in the study.

Sample	CS Dissolved in 2% AcAc [%]	CS Powder Suspended in Water [%]	WPI [%]
CSsol1	1	-	40
CSsol5	5	-	40
CSsol10	10	-	40
CSsol20	20	-	40
CSsol40	40	-	40
CSp20	-	20	40
CSp30	-	30	40
CSp40	-	40	40
CSp50	-	50	40
CSp60	-	60	40
WPI			40

## Data Availability

The authors confirm that the data supporting the findings of this study are available within the article.
